# The effective application of a discrete transition model to explore cell-cycle regulation in yeast

**DOI:** 10.1186/1756-0500-6-311

**Published:** 2013-08-06

**Authors:** Amir Rubinstein, Ofir Hazan, Benny Chor, Ron Y Pinter, Yona Kassir

**Affiliations:** 1 School of Computer Science, Tel Aviv University, Tel Aviv 69978, Israel; 2 Department of Mathematics, Ort Braude College, Karmiel 21982, Israel; 3 Department of Computer Science, Technion – Israel Institute of Technology, Haifa 3200003, Israel; 4 Department of Biology, Technion – Israel Institute of Technology, Haifa 3200003, Israel

**Keywords:** Cell-cycle, Commitment, Budding yeast, Computational model, Regulatory networks, Simulation

## Abstract

**Background:**

Bench biologists often do not take part in the development of computational models for their systems, and therefore, they frequently employ them as “black-boxes”. Our aim was to construct and test a model that does not depend on the availability of quantitative data, and can be directly used without a need for intensive computational background.

**Results:**

We present a discrete transition model. We used cell-cycle in budding yeast as a paradigm for a complex network, demonstrating phenomena such as sequential protein expression and activity, and cell-cycle oscillation. The structure of the network was validated by its response to computational perturbations such as mutations, and its response to mating-pheromone or nitrogen depletion. The model has a strong predicative capability, demonstrating how the activity of a specific transcription factor, Hcm1, is regulated, and what determines commitment of cells to enter and complete the cell-cycle.

**Conclusion:**

The model presented herein is intuitive, yet is expressive enough to elucidate the intrinsic structure and qualitative behavior of large and complex regulatory networks. Moreover our model allowed us to examine multiple hypotheses in a simple and intuitive manner, giving rise to testable predictions. This methodology can be easily integrated as a useful approach for the study of networks, enriching experimental biology with computational insights.

## Background

The fate of cells in response to changing signals is determined through regulatory networks [1]. The components, i.e. genes and proteins, are identified by experimental tools which also reveal interactions between these components. Computational modeling of these networks can help in elucidating their structure and properties, identifying missing components (designated nodes in computational models), and distinguishing between optional hypotheses regarding interactions (edges) between nodes. Computational models can be roughly described as either continuous, dynamic ones, or logical/Boolean ones [2-6]. The continuous models (either stochastic or employing differential equations) are detailed, yet are often computationally infeasible on a large scale, and require data such as kinetic constants or concentration levels, which are often unavailable. The Boolean models, on the other hand, are computationally efficient, but their expressive power is rather limited [7,8].

A richer, substantially more expressive yet computationally efficient logical approach, which is an extension of Boolean models, is a discrete transition model [7,8]. In the model suggested by Rubinstein et al. [7], each node assumes a non-negative initial state, which reflects its activity level at the onset of simulation. Regulation effects are represented by weighted edges, where positive or negative weights reflect activation or repression, respectively. Moreover, the effect of an edge can be subject to regulation by other nodes, reflecting essential dependencies between components. A uniform transition rule determines simultaneously how nodes' states change over time (which is also discrete). This model was applied for the study of entry into meiosis in budding yeast (an 8 node network), demonstrating the transient and sequential expression of its two master regulators [7]. Moreover, it was successfully used to discriminate between optional hypotheses, revealing missing regulatory elements that were subsequently identified using experimental tools [7].

In the present work, we refine our discrete model [7] by generalizing its state transition function. The resulting model is rich enough to describe the oscillatory behavior of the cell-cycle in the budding yeast S. cerevisiae (a much larger 66 node network); to distinguish among several optional hypotheses regarding a specific transcriptional regulator, Hcm1; and to predict the condition required for traversing START (restriction point) [9].

## Results

### Construction of the S. cerevisiae cell-cycle network

**Figure 1:**
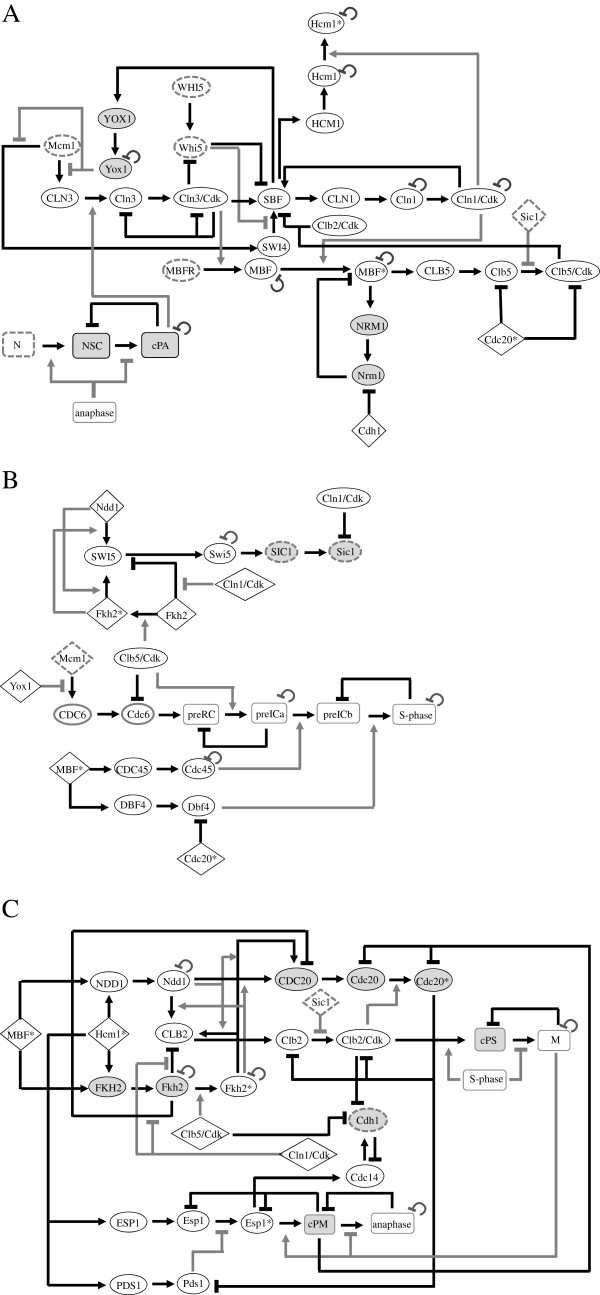
**A Schematic view of the cell-cycle in S. cerevisiae. A**. G1 and G1/S phases, **B**. S-phase, and **C**. G2 to anaphase. For simplicity we used this code to distinguish between the following: positive regulators – white ovals, negative regulators – gray ovals. Oval shapes with a dashed outline represent nodes with a constitutive state of 9 or whose initial state was 9. White diamonds represent regulators whose regulation appears in another part of the figure. Rectangles represent cellular events (white) and checkpoints (gray). Positive edges – arrows, negative edges – lines with bars, dependency edges – gray arrows from a node to an edge. Self-edges represent negative auto-regulation. Details on the construction of the network are given in Methods.

**Figure 2:**
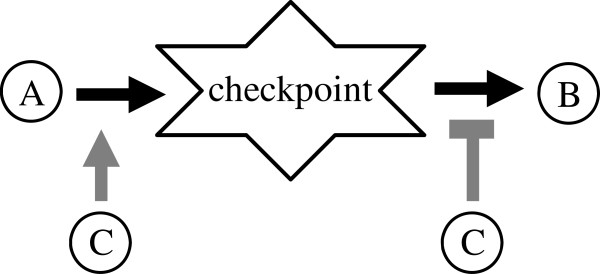
**A representation of a biological checkpoint.** The positive state of node **C** is a condition for **A** to activate the checkpoint node. The activation of **B** depends on the absence of **C**.

We used the budding yeast cell-cycle as a paradigm for a complex biological regulatory system. Figure 1 shows a schematic representation of this network. For details see Methods. The network includes 67 nodes which represent important components (i.e. RNA, proteins, cellular events) required for proper transitions between all cell-cycle phases. Redundant gene functions were represented in our network each by a single element. A checkpoint was modeled by a node whose level is induced by a specific regulator, but its ability to activate some other element depends on the absence of that regulator (Figure 2). For instance, the DNA replication checkpoint (cPS) is activated by S-phase, and it promotes metaphase only in the absence (completion) of S-phase [10] (Figure 1B).

### The discrete transition model exhibits oscillatory (periodic) and sequential behavior

**Figure 3:**
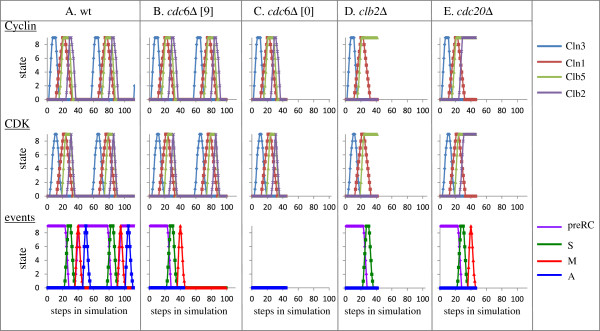
**Simulation of the network under normal conditions of wild type and mutant strains. A**. wild-type. **B**. and **C**. cdc6Δ. The initial states of CDC6 and Cdc6 are either 9 **(B)** or 0 **(C)**. **D**. clb2Δ, **E**. cdc20Δ. In **A** oscillations continued infinitely, whereas in **B-E** simulation reached steady state and all steps are shown.

The initial states assigned to the nodes reflect a single cell at early G1 (see details in Methods). In general, a simulation goes on until either a steady state or an infinite loop is reached. Our simulation demonstrated oscillation, accurate and sequential progression through S-phase, entry into metaphase (M), and exit from metaphase (anaphase, A) (Figure 3A). Figure 3A shows two cell-cycles, but identical oscillations occurred infinitely (data not shown). Our simulation revealed the sequential and periodic expression of the G1, G1/S, S and M-phase cyclins, namely, Cln3, Cln1, Clb5, and Clb2 (RNA and proteins) and their activities (when in complex with Cdk1) (Figure 3A), as expected from experimental results (reviewed in [11,12]). Periodic and timely expression was also evident for all transcription factors that regulate the cell-cycle (Additional file 1), in agreement with experimental data. [13]. This validates the network structure and parameters.

We used our model to predict the behavior of cells in the absence of specific regulators (Figure 3). Gene deletion was simulated by excluding outgoing edges of the deleted genes. DNA replication depends on CDC6, which is essential for preRC formation [14]. Our simulation predicts that exclusion of CDC6 will result in cell-cycle arrest prior to S-phase entry (Figure 3C), in agreement with reported data [15]. Cdc6 is loaded on origins at telophhase [16,17]. Indeed, when the initial states of CDC6 RNA and protein were 9, reflecting the normal level at early G1 cells, cell cycle arrest prior to DNA replication was not immediate, and occurred only at the subsequent cell cycle (Figure 3B). This is in agreement with the results reported when unsynchronized cdc6-ts cells were shifted of to the non-permissive temperature [18]. Note that these cells showed the expected transient expression and activity of the cyclins and their corresponding Cdks, and then arrested without these cyclins (Figure 3, B and C. In B cells reached a steady state without cyclins at step 105). Excluding CLB2 (representing CLB1 and CLB2) outgoing edges predicted an arrest after completion of DNA replication, prior to entry into M-phase (Figure 3D). In accord, experimental results demonstrated that Cdk/Clb1,2 are indeed required for entry into M-phase [19]. Exclusion of CDC20 resulted in a cell-cycle arrest prior to entry into anaphase, with high levels of Clb2 (Figure 3E). This prediction was confirmed, as CDC20 is required [in a complex with APC/C [20]] for exit from metaphase [21]. Since Clb2/Cdk is required to activate Cdc20 [22], its exclusion also caused an arrest with high levels of Clb5 (Figure 3E) whose degradation depends on Cdc20 [23]. In conclusion, the simulations of the various mutants gave rise to predictions that were confirmed by wet experiments.

### Cell-cycle commitment

Entry into the cell-cycle depends on external and internal signals. Within G1 there is a specific point designated START or restriction point, after which cells become committed to the cell-cycle, and will complete it even in the absence of a signal [9]. In order to examine how single cells, at different cell-cycle stages, respond to perturbations, we conducted simulations with initial nodes’ states that were reached in various intermediate steps of the normal conditions simulation. These initial states represent cells in G1, G1/S, S, M and A. We wished to use our model to determine how cells respond to α-factor or nitrogen depletion, and either arrest immediately in G1, or following completion of a cell-cycle.

**Figure 4:**
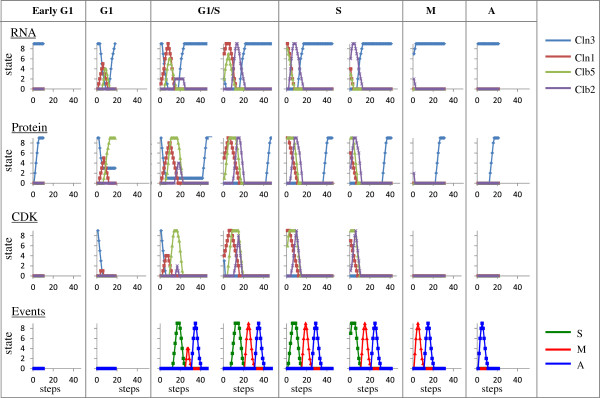
**Simulation in the presence of α-factor.** Cells at different steps in the normal cell-cycle simulation were “shifted” to simulations in which α-factor node is at state 9. Early G1 cells were taken from step 9, G1 cells from step 17, G1/S cells from steps 18 and 23, S-phase cells from steps 29 and 33, M-phase cells from step 43, and A-phase cells from step 53. Results regarding Cyclins (RNA and proteins), CDK activities and cell-cycle events are shown. All simulations reached steady state, and plots end at this step.

**Figure 5:**
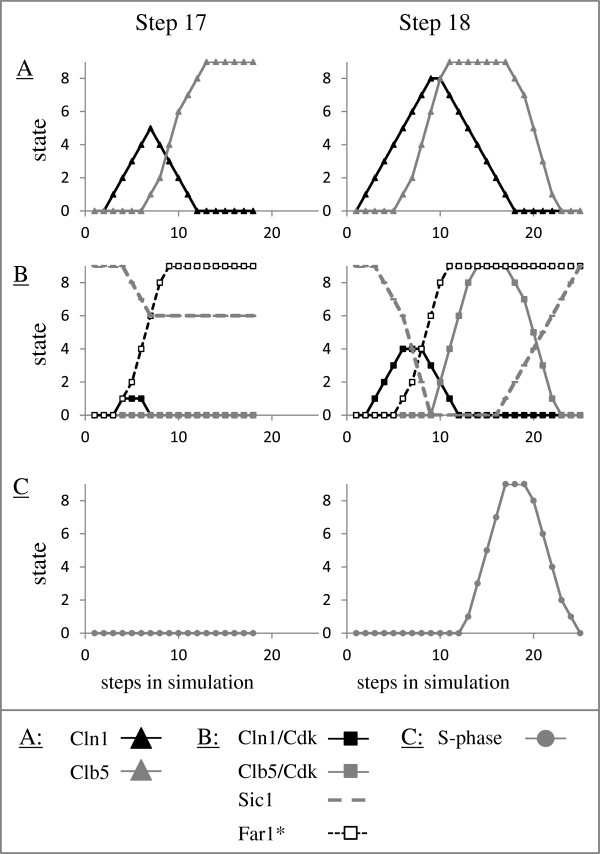
**Commitment to the cell cycle is determine by the level of Cln1/Cdk.** Simulation in the presence of α-factor as in Figure 4. **A**. Cln1, Clb5, **B**. Cln1/Cdk, Clb5/Cdk, Far1*, Sic1, **C**. S-phase.

Treatment with α-factor leads to inhibition of Cln3/Cdk and Cln1/Cdk functions [24-26]. Simulations showed that treatment with α-factor resulted in cell cycle arrest as a steady state was reached. Cells in which Cln3/Cdk was not yet active (early G1) exhibited immediate cell-cycle arrest prior to the transcription of the G1 cyclin CLN1 and entry into S-phase (Figure 4), as reported [27-29]. Cells that were already in S-phase, M-phase or anaphase completed the cycle and arrested in G1, with high levels of CLN3 RNA and protein, but CLN1, CLB5 and CLB2 RNA and proteins were absent (Figure 4). Commitment to the cell-cycle, namely entry into S-phase, occurred only in cells in which the activity of Clb5/Cdk was induced (Figure 4, compare G1 to G1/S cells). Note that at the onset of simulation Clb5/Cdk was not active in both G1 and G1/S (Figures 4, and 5B), although the Clb5 protein was induced. Since the activity of Clb5/Cdk is inhibited by Sic1 [30], we examined its level in these cells. Cells able to activate Clb5/Cdk showed a transient elimination of Sic1 (Figure 5B, step 18). In contrast, cells that were shifted to pheromone one step earlier, exhibited only a decline in the level of Sic1 (Figure 5B, step 17). This result points to Sic1 as the indicator for commitment, as previously suggested [31,32].

We further examined if the commitment actually depended directly on Sic1 or actually on its upstream regulator. Degradation of Sic1 depends on Cln1/Cdk [33]. In accord, the level of Cln1/Cdk was higher in cells able to complete the cycle, in comparison to cells that showed immediate arrest (Figure 5B, compare cells switched at step 18 and step 17). Thus, the low level of Cln1/Cdk (node at state 1 enabled only a decline in Sic1 state (Figure 5B, step 17). On the other hand, when Cln1/Cdk level reached the higher state of 4, Sic1 was completely degraded (Figure 5B, step 18). This analysis suggests that the ability of Cln1/Cdk to destabilize Sic1 defines the point of no return, namely traversing through “Start”, and that a threshold state/level of Cln1/Cdk is required for this effect. In agreement, using single-cell analysis, it was concluded that, the induction of Cln1/2 feedback, which results in higher activity of Cln1/Cdk, provides a biochemical definition for Start [34].

Under normal conditions the level of Cln1 mirrors the level of Cln1/Cdk (Figure 3A). On the other hand, following pheromone treatment, the level of Cln1/Cdk is lower than expected (Figures 5A and 5B). This suggests that an additional factor plays an important role in cell-cycle commitment. Far1* (the active, phosphorylated form of Far1) inhibits Cln1/Cdk function [24], whereas Cln1/Cdk destabilizes Far1 [35]. We examined, therefore, the effect of Far1* on the level of the Cln1/Cdk. Cells switched at step 18 showed a delay in the increase of Far1* in comparison to the time of induction of Cln1/Cdk (Figure 5B). On the other hand, cells switched at stage 17 showed an earlier induction of Far1*, at the same time as that of Cln1/Cdk (Figure 5B). We conclude, therefore, that Cln/Cdk activity defines commitment, and that under pheromone induction this activity is regulated by the double negative feedback loop between Cln1/Cdk and Far1*, as previously suggested [34].

Nitrogen depletion leads to G1 arrest [36]. It is assumed that its main target is the G1 cyclin, CLN3, as nitrogen starvation inhibits CLN3 mRNA translation [37], increases Cln3 protein degradation [37], and retains Cln3 in the cytoplasm [38]. It was speculated that nitrogen depletion has an additional target, since cells that are deleted for CLN3 properly arrest in G1 following nitrogen depletion. Our simulation reinforces this speculation, as nitrogen depletion promoted cell-cycle arrest, mainly in G1, but a specific subpopulation arrested in G2, after completion of DNA replication and prior to entry into M-phase (Table 1, hypothesis 1)). This result suggests that indeed nitrogen depletion must affect an additional regulator. Two possible targets were previously suggested: SIC1 mRNA availability (hypothesis 2) or Cln1/2 stability (hypothesis 3) [37]. Simulations of these two hypotheses resulted in the correct G1 arrest (Table 1). In order to discriminate between these two hypotheses we conducted simulations of a strain expressing a stable Cln1 protein. This approach was based on the report that cells which express a stable Cln2 protein (CLN2-1 allele) respond to nitrogen depletion by arresting the cell-cycle at multiple points, not only in G1 [39]. Simulation of hypothesis 2 network resulted in the arrest of all cells in G1, whereas simulation of hypothesis 3 network resulted in the correct behavior, namely arrest in both G1 and G2 (Table 1). Our results predict, therefore, that nitrogen depletion also affects the stability of Cln1.

**Table 1:** Simulations predict that nitrogen depletion affects both Cln3 and Cln1

**Hypotheses**	**Possible nitrogen depletion targets**	**Results of simulations**	
		**Point of cell-cycle arrest**	
		**Wild-type**	**Stable Cln1**
Hypothesis 1	Cln3, Cln3/Cdk	G1 and G2	G1 and G2
Hypothesis 2	Cln3, Cln3/Cdk, SIC1 transcription	G1	G1
Hypothesis 3	Cln3, Cln3/Cdk, Cln1, Cln1/Cdk	G1	G1 and G2

Cells at different steps in the normal cell-cycle simulation were “shifted” to simulations in which Nitrogen was depleted.

### The use of our model to predict the Cdk/cyclin complex that regulates Hcm1

**Figure 6:**
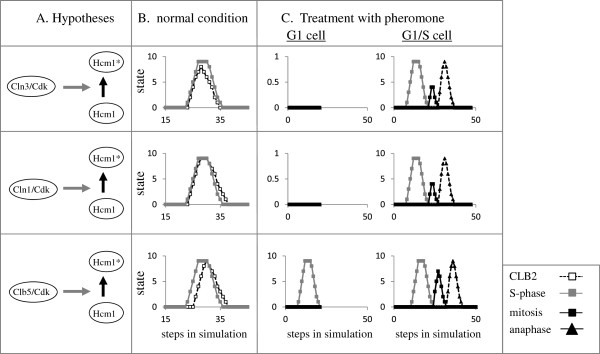
Hypotheses regarding how Hcm1* activity is regulated. A. hypotheses; **B**. levels of CLB2 and S-phase; **C**. levels of S-phase, mitosis and anaphase in α-factor treatment of cells taken from G1 and G1/S stages.

The transient expression of Hcm1 is not required for the transient transcription of its target genes [13]. Because Hcm1 is subject to post-translational modification, it was suggested that this modification affects its activity during the cell cycle [13]. Since Hcm1 is a probable Cdk target [40] we examined if this regulation is mediated by either Cln3/Cdk, Cln1/Cdk1 or Clb5/Cdk (Figure 6). Simulations revealed that activation of Hcm1 by Cln3/Cdk resulted in premature decline in the transcription of CLB2 in relation to S-phase (Figure 6B, upper panel). On the other hand, regulation by either Cln1/Cdk or Clb5/Cdk, showed the expected behavior (Figure 6B, middle and lower panels). In order to discriminate between the latter two hypotheses, we examined the response of cells to pheromone treatment. Regulation by Clb5/Cdk showed an abnormal phenotype, namely some G1cells arrested after completion of S-phase (Figure 6C). Our simulations predict that Cln1/Cdk rather than Clb5/Cdk or Clb2/Cdk, is responsible for regulating the activity of Hcm1. All simulations in this report were done according to this prediction.

## Discussion

The analysis of regulatory networks by computational methods is often quantitative in nature. However, due to lack of complete quantitative kinetic data in many cases, these methods are not applicable. Moreover, the use of these methods requires intensive computational skills, and are typically applied by either trained bioinformaticians or employed in a collaborative, interdisciplinary manner. Experimental biologists, by themselves, rarely incorporate these methods in their routine research, and often refrain from reviewing the computational analyses in scientific literature. Consequently, manuscripts which include intense use of mathematical equations are less frequently cited by experimental biologists [41]. Furthermore, the use of existing bioinformatics tools by scientists who lack intensive background in bioinformatics may result in misinterpretation of simulation results and in erroneous conclusion making [42].

In this report we demonstrate that a simple discrete model can suffice for the qualitative analysis of important network properties, but still remain intuitive for use without extensive computational background. Indeed, our results could not have been reached without a repeated, intuitive, refinement process, made of numerous simulations conducted directly by a biologist. This type of model is a “middle ground” between Boolean methods, which are claimed to gradually fall out of favor [4], and quantitative models. Additional file 1 describes the simple mode by which interested scientists can apply this model, using the tool we implemented. As described, this process requires only a straightforward conversion of a biological network into a specified format.

We applied our model to the yeast cell-cycle network demonstrating cell-cycle oscillation (Figures 3 and Additional file 1). The network included 67 nodes, 60 of which showed the reported sequential and periodic expression (the other 7 were either constitutively present or active only under pheromone treatment) (Figures 3 and Additional file 1). Thus, the model effectively demonstrated the correct behavior of a fairly large and non-linear network. Predictions showing an arrest at three essential points: prior to entry into S-phase, metaphase, or anaphase (Figure 3) were confirmed by published data.

Entry of eukaryotic cells into alternative differentiation pathways is usually executed at G1 [36] Thus, signals that regulate these developmental pathways will first lead to arrest in G1 and subsequently to entry into the new developmental pathway. Consequently, within G1 there is a point, START, prior to which cells will respond by immediate arrest at G1, whereas cells that passed this point are committed to complete the cell-cycle and only then arrest in G1. In this report, using our discrete model, we examined how yeast cells respond to either the mating (pheromone) or nitrogen depletion signals. We show that as reported, cells are divided into pre- and post- START (Figure 4). Pre-START cells showed an immediate arrest, whereas post-START cells where committed, completed the cell cycle, and then arrested in G1 (Figure 4 and Table 1).

Simulation in the presence of pheromone allowed us to identify the component whose expression/activity defined START. We showed that Cln1/Cdk expression is required for commitment. Moreover, Cln1/Cdk level was important, as a low state of 1 for that node did not suffice for traversing the cell cycle. Finally, our results point to the double-negative feedback loop between Cln1/Cdk and Far1* as an important parameter in regulating the level of Cln1/Cdk, and consequently the commitment stage (Figure 5), reinforcing a previous conclusion that was based on experimental results and modeling by differential equations [34].

Simulation under nitrogen depletion allowed us to predict the targets for this signal. We showed that an effect only on Cln3 did not suffice for an arrest of all cells in G1, and that in addition, starvation also affected the availability of Cln1 (Table 1). Moreover, our simulations refute the hypothesis that SIC1 is an essential target of nitrogen depletion (Table 1). This demonstrates the predictive capability of our model. We also examined 3 hypotheses regarding the regulation of Hcm1 activity. The model predicted that Cln1/Cdk, rather than Cln3/Cdk or Clb5/Cdk, mediates this regulation (Figure 6). In conclusion, our model allowed us to examine multiple hypotheses in a simple and intuitive manner, giving rise to testable predictions.

The number of nodes used, 67, in this study does not represent the complete cell-cycle regulatory network. Our network (Figure 1) can be used as a starting point for an in-depth, focused analysis of specific cell-cycle events. For instance DNA-replication, G2-M transition, exit from metaphase, or checkpoint regulation. Our model is available upon request.

## Conclusions

We present a simple and intuitive model that does not depend on the availability of quantitative data, and can be directly used without a need for intensive computational background. This methodology can be easily integrated as a useful approach for the study of networks, enriching experimental biology with computational insights. The validity of the model was tested on a large and complex network, cell cycle in budding yeast. We verified the structure of the cell cycle network by simulations of various mutants. The model has a strong predictive feature that can be easily used to distinguish between alternative hypotheses. Herein the model was used to predict the following: 1. Cln1/Cdk rather than Cln3/Cdk, Clb5/Cdk or Clb2/Cdk, is responsible for regulating the activity of Hcm1, and (2). Simulations in the presence of α-factor predict that commitment to enter the cell cycle depends on a double-negative feedback loop between Cln1/Cdk and Far1*.

## Methods

### The computational model

Our model is an extension of the discrete transition model suggested by [7]. In this model, nodes represent mRNA's, proteins, nutrients, or cellular events. Each node assumes an initial discrete state taken from a fixed range {0,…,U} [e.g. U=9, as in our simulations, chosen for technical reasons, see [7]], and this state may change over time. Edge (i,j) acquires a positive (activation) or negative (repression) weight w(i,j). “Dependency edges”, going from some node k to an edge (i,j), introduce dependent regulation effects: In order for i to regulate j, node k must be active (positive dependency) or inactive (negative dependency). A configuration of the system is a vector of all the nodes’ states. A transition function determines the next state of each node, given its current state, the states of its neighbors and the weights of its incoming edges. A simulation step is an application of the transition function simultaneously to all nodes in the system. Steps occur in discrete times t = 1, t = 2 and so on. A simulation is a consecutive application of the transition function forming a sequence of steps, starting from a designated initial configuration, and continuing until either a steady state is reached (two consecutive identical configurations), or an infinite loop of configurations is detected.

We extend the model's transition function, which appears in Formula 1. s_i_(t) is the state of node i at time t. The term sum_i_(t) captures the total effect on node i at time t by all its neighbors. We remark that *cond*(*j*, *i*) = 1 if all dependency conditions on edge (j,i) hold, and 0 otherwise. If sum_i_(t) exceeds the upper threshold of node i (threshold_i_^+^) its state will increase in the next time step, while if it is below the lower threshold (threshold_i_^-^) its state will decrease. The update is (in the first two cases) some function f of sum_i_(t). In [7], *f* = 1, whereas we introduced a logarithmic-order transition function, *f*(*sum*_*i*_(*t*)) = [ln(|*sum*_*i*_(*t*)| + 1)]. We note that the logarithmic function avoids the problematic nature of, e.g., linear functions, which cause changes which are too extreme, and of constant change functions, that are not sufficiently differential. This enriched model enables the transition function to reflect differential strengths of regulation effects, such as different elements showing faster increase or decrease of activity, compared to others.

### Formula 1: The extended transition function

$$\begin{array}{l} s_{i}\left(t+1\right)=\left\{\begin{array}{c} \min\left(U,s_{i}\left(t\right)+f\;\left({\text{sum}}_{i}\left(t\right)\right)\right)\\ \max\left(0,s_{i}\left(t\right)-f\;\left({\text{sum}}_{i}\left(t\right)\right)\right)\\ s_{i}\left(t\right) \end{array}\;\begin{array}{c} \text{if}\;{\text{sum}}_{i}\left(t\right)>{\mathrm{threshold}}_{i}^{+}\\ \text{if}\;{\text{sum}}_{i}\left(t\right)<{\text{threshold}}_{i}^{-}\\ \text{otherwise} \end{array}\;\right\}\\ \text{where}\;{\mathrm{sum}}_{i}\left(t\right)=\sum_{j}w\left(j,i\right)\cdot s_{j}\left(t\right)\cdot \mathrm{cond}\left(j,i\right) \end{array}$$

The model was implemented in C# using Visual Studio.NET, and analyses procedures of simulation results were implemented as VBA macros.

### Construction of the cell cycle network

The following general considerations were used to construct the network (Figure 1). Redundant gene functions were represented in our network each by a single element. Periodic availability/activity requires that each node is subject to both positive and negative inputs. RNAs are non-stable molecules, nonetheless in most cases there is no information regarding the control of mRNA stability. Therefore, in the network, we simulated the intrinsic stability of the RNAs by using negative auto-regulation. In many cases, reported results demonstrated periodic expression of proteins, however, only in few cases information was reported regarding how these proteins’ stability is regulated. Therefore, in order to simulate this behavior we either used negative auto regulation, or negative feedback regulation. Some proteins are represented by two nodes: unmodified and modified (labeled with a star). Essential regulatory elements are present as dependencies edges.

The information used to construct the cell cycle network is described below. The G1 and G1/S transition (Figure 1A): Entry into the cell cycle from G1 depends on both the availability of nutrients (N) and the completion of anaphase. The major regulator is Cln3/Cdk1 (Cdk1is designated inhere as Cdk). Cdk expression is constitutive, while the transcription of CLN3 is regulated by Mcm1, a constitutive expressed TF (level 9 throughout the simulation). Mcm1 activity is repressed by Yox1 [43] (Yox1 regulation will be described below). The translation and stability of Cln3 protein is subject to multiple regulations by nutrients [37,44,45]. We assumed that depending on anaphase, nutrients regulate a nutrient sensing node (NSC), and that this node regulates a checkpoint (cPA) whose availability depends on the completion (absence) of anaphase. We further assumed that the translation of Cln3 depends on this checkpoint. Finally, Cln3 is degraded following phosphorylation by Cdk [46]. We assume that this regulation is mediated through the Cln3/Cdk complex. Cln3/Cdk promotes the activity of two transcription factor complexes, SBF and MBF. SBF represses transcription when it consists of Swi4/Swi6/Whi5, and activates transcription when Whi5 dissociates from the complex. The transcription of WHI5 is regulated by Hcm1* [13]. However, because in cells deleted for HCM1 its transcription is constitutive, but less than the wild type level [13], we omitted regulation by Hcm1*, and designated its regulation as constitutive, with initial level of 5. The activity of Whi5 is negatively regulated by Cln3/Cdk, an event that causes its dissociation from the complex [47]. In order to prevent entry into the cell cycle until Cln3/Cdk is available, the initial state of Whi5 was 5.

The transcription of SWI4 is regulated in the same manner as that of CLN3 [43]. In the network SWI4 regulates SBF formation. For simplicity, we did not separate SBF into its repression and activation complexes. Instead, the essential repression functions of Whi5 [48,49] was modeled as a dependency edge that inhibits the ability of Swi4 to activate SBF, as well as by a direct negative regulation on SBF. In addition, the function of SBF is positively regulated by both Cln3/Cdk and Cln1/Cdk [29,50]. SBF function is negatively regulated by Clb2/Cdk [51] as well as by Clb6/Cdk, as phosphorylation of Swi6 by Clb6/Cdk leads to the export of Swi6 from the nucleus to the cytoplasm. Dephosphorylation of Swi6 by Cdc14 promotes nuclear import, in preparation for a new cell cycle [52]. This effect is designated in the network as an edge from Clb5/Cdk.

The transcription of YOX1 is periodic, regulated by SBF [13,43]. Both SBF and MBF bind to the HCM1 promoter [53]. These transcription factors have apparently redundant functions, because deletion of both TFs was required in order to observe an effect on its transcription [54]. The network includes regulation by only one complex – SBF, which is functional prior to MBF. The Hcm1 protein shows periodic expression [13], we assume that this is due to its intrinsic stability, similarly to Yox1. Hcm1 is switched to the active Hcm1* depending on Cln1/Cdk (see rational in Figure 6 and text).

The transcription of CLN1 is regulated by SBF [49,55]. Expression of Cln1 is periodic; we assume that this is due to Cln1 intrinsic stability.

MBF is detected on promoters throughout the cell cycle, although at most times it represses transcription [56]. Transcriptional activation by MBF depends on both Cln3/Cdk and Cln1/Cdk. Therefore, we divided this complex into 3 nodes: MBFR (for repressive), MBF, and MBF* (for active). MBFR level is constitutive 9. MBFR is switched to MBF depending on Cln3/Cdk, whereas MBF is switched to MBF* depending on Cln1/Cdk. The level of MBF is decreased depending on self-degradation. Activity of MBF* is inhibited by Nrm1 [56]. In cells deleted for NRM1 the transcription of MBF targets is still periodic [56], and because we do not know who is responsible for this effect, we put self-degradation on MBF*.

The transcription of NRM1 is regulated by MBF* [56]. The periodic expression of Nrm1 is mediated by degradation via Cdh1/APC [57]. MBF* regulates the transcription of CLB5. The periodic expression of Clb5 and Clb5/Cdk is mediated by degradation from APC/C/Cdc20* [21,23]. The level of APC/C is constitutive, but its activity depends on its association with either Cdh1 or Cdc20 [20]. Therefore, in our network, Cdc20* represents APC/Cdc20. In addition, stability of Clb5 depends on an additional proteasome depending factor whose identity is still not known [58]. As this unknown regulation is absent from our network, the level of Clb5 was not reduced to 0 upon treatment with pheromone. Finally, the activity of Clb5/Cdk depends on the absence of Sic1 [30].

Entry into S-phase (Figure 1B): DNA replication is a complex process that depends on many proteins. In the network presented in here we used only few proteins that suffice to define its separation to distinct phases/complexes. Below we first describe the regulation of these proteins and then how they are used to regulate DNA replication. The transcription of CDC6 is positively regulated by Mcm1 depending on the absence of Yox1 [43]. The periodic availability of Cdc6 is accomplished through negative regulation from Clb5/Cdk [59,60]. Cdc6 is present prior to G1, and therefore, the initial levels of CDC6 and Cdc6 were given the state 9. The transcription of CDC45 and DBF4 are positively regulated by MBF* [53,61]. Cdc45 availability is regulated by intrinsic stability. In order to shorten and control time of expression, the positive and negative regulations edges were assigned the weight 2. The stability of Dbf4 is regulated by APC/Cdc20 [62]. Pre-Replication Complex (preRC) formation is regulated by Cdc6 [14]. The preRC complex was switched to PreIC depending on Clb5/Cdk1 [62,63]. The switch from preIC to preIC* depends on Cdc45 [62,63]. To simulate the switch from preRC to preIC, preRC was negatively regulated by preIC, and preIC was eliminated by self-degradation. Finally, entry into S-phase from preIC* depended on the function of Dbf4 [64]. Both preIC* and S-phase were terminated by a negative feedback loop from S-phase.

The G2 to anaphase transition (Figure 1C): Hcm1* regulates the transcription of both NDD1 and FKH2 [13]. However, in cells deleted for HCM1 transcription remained periodic with a shift in peak time, indicating a combinatorial control by both Hcm1 and at least one other cell cycle-specific regulator that promotes transcription later in the cycle [13]. Because there is a non-perfect site for MBP in both NDD1 (at −556 ACGCGc instead of ACGCGT) and FKH2 (ACGCtT at −530), in the network regulations by both Hcm1* and MBF* were added. We assume that the periodic expression of Ndd1, Fkh2 and Fkh2* are due to the intrinsic stability of the proteins. Fkh2 that functions as a negative regulator is converted into a positive one, designated Fkh2*, depending on phosphorylation by Clb5/Cdk [65].

The transcriptions of CLB2, SWI5 and CDC20 are negatively regulated by Fkh2, depending on the absence of Cln1/Cdk activity [66]. Transcription is positively regulated by Ndd1 depending on Fkh2* (which recruits Ndd1 to the promoter). Transcription is also positively regulated by Fkh2* depending on Ndd1 [65,67,68]. The transcription of CLB2 is initiated following entry into S-phase, and is completed following the completion of S-phase [19]. The use of a limited number of regulators in our network resulted in earlier expression of CLB2. In order to overcome this effect, we delayed the transcription of CLB2 by putting an upper threshold of 8 on its positive regulator – Fkh2*.

The periodic expression of Clb2 is regulated by Cdc20/APC [21,23]. The activity of Clb2/Cdk depends on the absence of Sic1 [21]. Swi5 level is periodic [69], but since its mode of regulation is not known, we assumed intrinsic stability. Activation of Cdc20, designated Cdc20* is via phosphorylation by Clb2/Cdk [22,70]. We assume that Cdc20 and Cdc20* availability are regulated by a negative feedback from cPM (checkpoint M).

The transcription of SIC1 is positively regulated by Swi5 [71]. The stability of Sic1 is regulated following phosphorylation by Cln1/Cdk [33]. In order to delay entry into S-phase until cells express Cln1/Cdk, in the simulation the initial state of SIC1 and Sic1 was 9.

Entry into metaphase: (Figure 1C): Entry into metaphase depends on a checkpoint that monitors the completion of S-phase. This checkpoint was represented in the following simplified manner: We assume that Clb2/Cdk activates the S checkpoint (cPS) depending on S-phase. This checkpoint activates entry into metaphase depending on the completion (absence) of S phase [19]. We assigned the weight of that edge to 2, because in our simplified network many regulators required for entry into metaphase are missing, and thus the use of a single edge was unable to promote an increase of the metaphase node to 9, whereas the use of level 2, sufficed. Down regulation of cPS is by metaphase, and metaphase is subject to negative auto-regulation.

The transcription factor that regulates the transcription of PDS1 is not known. Transcription profile resembles CLN1 transcription, however, neither SBF nor MBF bind to PDS1 promoter [53]. We assume that it is regulated by Hcm1* because it carries a consensus for its binding, atAAACAAa at −148 [consensus is AAAAACAAA [13]]. Protein availability is regulated by Cdc20*/APC [72]. The transcription of ESP1 is regulated by Hcm1* [13]. Esp1 is inactive in the presence of Pds1 [73]. We represent this regulation by the addition of an active Esp1 node whose presence depends on the absence of Pds1. We do not know how Esp1 and Esp1* availabilities are regulated; we assume negative feedback from cPM.

The activity of Cdh1 is positively regulated by Cdc14 and negatively regulated by both Clb2/Cdk and Clb5/Cdk [74]. The activity of Cdc14 is positively regulated by Esp1* and negatively by APC/Cdh1 [75].

Entry into anaphase also depends on a checkpoint. We assumed that this checkpoint (checkpoint M – cPM) is activated by Esp1* depending on entry into M-phase. Entry into anaphase is activated by this checkpoint depending on the completion of metaphase. In the network only limited number of regulators that activate anaphase were used, therefore, in order for anaphase to reach a maximal state of 9, we used edge weight of 2. Completion of both the checkpoint and anaphase is under negative feedback regulation from anaphase.

Regulation by pheromone: Under normal conditions the level of pheromone (excluded from Figure 1) was 0, while for the mating pheromone response its level was 9. Pheromone treatment results in inhibition of Cln3/Cdk, and Cln1/Cdk activity [26]. Our network does not include most of the details on how this signal is transmitted. Inhibition of Cln1/Cdk was mediated by Far1*. Pheromone regulates Far1* in two modes: Firstly, in response to pheromone treatment the transcription of FAR1 is induced [76], and the protein is activated following phosphorylation [24]. Finally, phosphorylation of Far1 and Far1* by Cln1/Cdk tags it for degradation [35].

The initial state of Nitrogen depletion signal was 9, and it was represented as N. This node repressed Cln3 along with Cln3/Cdk, and either Cln1 along with Cln1/Cdk, or SIC1, according to the hypotheses examined (see Table 1 and text).

## Competing interests

The authors declare that they have no competing interests.

## Authors’ contribution

AR and OH implemented the computational model. YK constructed the network and conducted simulations. AR and YK analyzed simulations results. AR, YK, RP and BC designed research methodology. AR and YK wrote the manuscript. All authors gave intellectual input and comments on the manuscript. All authors read and approved the final manuscript.

## Supplementary Material

Additional file 1 Tool manual. Click here for file
