# Supplementary material for: The effective application of a discrete transition model to explore cell-cycle regulation in yeast
Source: BMC Res Notes. 2013 Aug 6;6:311. doi: 10.1186/1756-0500-6-311 (PMC3750494; doi:10.1186/1756-0500-6-311)
Supplement: Additional file 1 — Tool manual. [file 1756-0500-6-311-S1.docx]

This document describes how the computational tool is applied.

The first part is a general guideline, and the second part gives a simple example.

**Part A**

**Installation**

The installation file can be sent upon request to the authors (amirr@tau.ac.il).

**Tool handling**

There are three operations a user can invoke: updating an existing input file, running a simulation, and analyzing results.

Input is given to the simulator as a Microsoft Excel file. The input file should be given in a specific format, described below. To avoid format errors, there is no option to create a new input file from scratch, only to update an existing one. Therefore, the installation comes with template input files, which should be the starting point for a newly created input file. Copy the template file and give it a new name, then use the "update existing input file" option.

Since networks may vary in number of nodes, we have created several template files, corresponding to different network sizes. For example, a user who intends to work with around 10 nodes may find it easier to work with a template of that size, rather than a template with a much larger number of nodes. These template files are located in the folder specified during the installation process, under "\UserFolders\Input Templates", and their names begin with "Basic Format…". The full names of the files indicate the maximal size of the network supported by each template. However, this is only for convenience: the user can choose to work with any template whose size is at least the number of nodes in the network.

1. **Updating an existing input file**

Choose which Excel file you want to update as input, and open it. The format of the input is as follows:

- - - In cell D3 enter the value for *U*, the maximal state a node can assume. This value must be an integer between 1 (Boolean model) and 15. For most cases consider *U*=9 as the appropriate value.
    - Names of nodes in the network should be entered only in the topmost row of the first matrix (cell B3 and right bound). The leftmost column of this matrix, as well as all other topmost rows and leftmost columns in the sheet are updated automatically.
    - The first matrix is a weight matrix. Enter the weight of edge from node X to node Y in the row named X and the column named Y. A weight can be any real number (positive or negative).
    - The second matrix specifies the dependency (*cond*) edges. In the cell corresponding to edge from X to Y, enter the sequential number (starting from 1) of the node that edge X🡪Y depends on. If the dependency is positive, enter it as a positive index, otherwise as a negative index. For example, if the edge X🡪Y depends negatively on node Z, which is the 7^th^ node in the list, enter -7 in the cell located in row X and column Y.
    - The next matrix is for the initial states of nodes. For each row, enter the initial states of the corresponding node. Initial states must be in the range [0,…,*U*]. At least one initial state should be given for every node, and obviously at most *U*+1.
    - The last part of the form specifies the lower and upper thresholds each node assumes. The default for both thresholds is 0. The lower threshold (*threshold_i_*^-^) should be a non-positive real number, and the upper threshold (*threshold_i_*^+^) a non-negative one.

Finally, save your updated input file in some location you will easily find when later running a simulation.

1. **Running a simulation**

First, choose the transition function, among the 3 options mentioned in the paper: constant, logarithmic and linear. This choice is made in the main window of the tool (not in the input file).

Then click the button for running the simulation. Choose the desired input file. A simulation will run in the background, resulting in a text file that opens with the simulation results. The name of that file is the same as the input file, except for the extension which is .txt and not .xls.

The results file first specifies all the simulation parameters given in the input file, to ease a later navigation between different result files. Then, all the initial vectors appear, each in a separate line, with the resulting trajectories (vectors reached step by step in the simulation). The final vector in each such line is either a steady state vector, or the last vector in a simulation that has not ended after 200 steps (an arbitrary value that specifies a condition to terminate the simulation, probably, but not necessarily indicating an infinite loop).

Last, the statistics of the simulation appear: the set of final vectors that were reached in the simulation, each with the percent of initial vectors that led to it, and the average and maximal length of the trajectories leading to it.

We suggest saving the results file in an easy-to-find location for a later analysis. However, the results file is also saved automatically in the folder "…\Plot Graphs BioV1.03\Temporary Files".

1. **Analyzing results**

This option allows plotting the profile of nodes in any single trajectory. Any results text file previously created can be the target of this option. An Excel file "Graph.xls" which is a part of the installation file will be opened.

- - - Click on the button "Load Results", and choose the results text file you are interested in.
    - Scroll down to the trajectories of the simulation.
    - Mark the whole row of the trajectory you want to analyze (by clicking on the number of the row).
    - Press CTRL+M.

A new sheet will open, containing a plot of the profiles of all the nodes in the chosen trajectories. This step may take a few seconds, especially when the number of nodes is large.

You can go back and choose another trajectory to plot.

**Part B**

The Figure below represents a simple network as an example for the creation of the excel file (the excel file is also attached).

You first write in row 3 the names of the nodes you have. These names will appear automatically in column A.

Node A positively regulates node B. You first need to decide how strong this regulation is. Usually, for simplicity (unless you know otherwise), the level you give is +1. You write it in the row designated A and in the column designated B. Similarly, B positively regulates both C and D, albeit at different levels, 1 and 0.5, respectively. Therefore in the row designated B you write +1 in column C, and +0.5 in column D. Finally, C negatively regulates A, and so -1 is inserted at the correct place.

The second part includes dependencies: B regulates C depending on the existence of D. Because D is the 4^th^ node in the excel file, you put the number +4 in the box that intersects row B with column C.

The third matrix gives the initial states of the nodes.

The last matrix consists two columns – negative and positive thresholds for each node (all are 0 in this example).

This Excel sheet is ready for simulation.
